# The Role of Prediagnosis Audiovestibular Dysfunction Versus Distress, Illness-Related Cognitions, and Behaviors in Predicted Ongoing Dizziness Handicap

**DOI:** 10.1097/PSY.0000000000000857

**Published:** 2020-08-27

**Authors:** David Herdman, Sam Norton, Marousa Pavlou, Louisa Murdin, Rona Moss-Morris

**Affiliations:** From the Health Psychology Section, Institute of Psychiatry Psychology and Neuroscience (Herdman, Norton, Moss-Morris), King’s College London; St George's University Hospitals NHS Foundation Trust (Herdman); Guy’s and St Thomas’ NHS Foundation Trust (Murdin); and Centre of Human and Aerospace Physiological Sciences (Pavlou), King’s College London, London, United Kingdom.

**Keywords:** dizziness, vestibular, handicap, anxiety, depression, illness perceptions, **BPPV** = benign paroxysmal positional vertigo, **DHI** = Dizziness Handicap Inventory, **GAD-7** = Generalized Anxiety Disorder-7, **IPQ-R** = Illness Perceptions Questionnaireâ Revised, **PHQ-9** = Patient Health Questionnaire-9, **SD** = standard deviation, **vHIT** = video head impulse test

## Abstract

**Objective:**

People with chronic vestibular diseases experience variable degrees of self-perceived disability. However, longitudinal data examining the predictive validity of relevant clinical variables alongside psychological variables are limited. The present study examined whether these factors predict self-reported dizziness handicap 3 months after assessment and diagnosis.

**Methods:**

Patients were recruited from a waiting list of a tertiary neuro-otology clinic and completed standardized mood, cognitive, behavioral, and dizziness handicap questionnaires before and 3 months after their initial consultation and diagnosis. All patients were clinically assessed and underwent comprehensive audiovestibular investigations.

**Results:**

Seventy-three percent of participants responded at follow-up (*n* = 135, 73% female, mean [standard deviation] age = 54.23 [17.53] years), of whom 88% were diagnosed with a neurotological condition. There were significant improvements in handicap, depression, and anxiety at 3 months. Thirty (22%) of 135 showed clinically meaningful improvement in handicap. The percentage of case-level depression and anxiety remained the same. Negative illness perceptions and symptom responses reduced, although participants still tended to view their condition negatively. Vestibular tests and type of diagnosis were not associated with self-reported handicap. Most baseline psychological variables significantly correlated with handicap at 3 months. When adjusting for baseline handicap and demographics, the baseline psychological variables only explained a significant ~3% of the variance in dizziness handicap at follow-up, with baseline handicap explaining most of the variance. All-or-nothing behavior was the most significant predictor.

**Conclusions:**

Tertiary patients with vertigo and dizziness report negative illness perceptions and cognitive and behavioral responses to symptoms that are associated with self-reported handicap over time. Future studies are needed to investigate whether targeting these factors alongside traditional treatment approaches improves handicap in patients with chronic dizziness.

## INTRODUCTION

The term “dizziness” refers either to a disturbance of spatial orientation or to a false perception of movement, which is more specifically called “vertigo” (1). Dizziness is a common complaint in medicine, and around 20% to 30% of people will experience rotatory vertigo (2–4), which may be interpreted as a more specific marker of vestibular disturbance. Vestibular disorders can also be associated with a wide range of physical symptoms such as unsteadiness, unstable vision, motion intolerance, and autonomic symptoms, as well as cognitive symptoms ranging from impaired spatial xlearning and memory to altered sense of body ownership and embodiment (5–7).

These symptoms can result in substantial morbidity and disability, especially in patients with chronic symptoms. One in 10 people of working age report some degree of handicap due to current dizziness (8). A significant proportion of people are sufficiently disabled or distressed to be referred for investigation and management to hospital outpatient clinics. In many patients, a structural vestibular disorder can be identified, although “functional” or “medically unexplained” dizziness syndromes can also occur as primary or secondary conditions (9).

The Dizziness Handicap Inventory (DHI) (10) has been widely adopted in specialist settings to measure self-perceived dizziness-related disability. There is substantial variability in the levels of handicap even in relatively homogenous patient groups (11). The level of handicap does not necessarily correlate with deficits on neuro-otological tests measuring the structural integrity of peripheral or central vestibular systems (12–14). In contrast, studies have shown strong correlations between the DHI and anxiety, depression, and autonomic arousal (15–21) and pathophysiological mechanisms have been proposed to explain this (22). Patients with prior anxiety and neurotic personality traits may also be more likely to develop secondary functional disorders such as “persistent postural perceptual dizziness” (23). Although premorbid and comorbid mental health issues seem to play a role, the evidence to date suggests that they cannot fully explain the extent of the dizziness handicap. Not all patients have mental health disorders, and developing therapeutic treatments based on models of anxiety may be suboptimal (24).

A handful of other studies have explored the role of patients’ emotional responses to symptoms and beliefs about their illness in perpetuating handicap and dizziness symptoms. In an early study, Yardley et al. (17) found that negative beliefs about the consequences of dizziness including fear of losing control were a significant predictor of dizziness and disability levels over time. Yardley et al. (25) also found that beliefs about the negative consequences of dizziness at baseline predicted handicap for 6 months and could be effectively reduced with vestibular rehabilitation. Follow-up studies of patients with acute vestibulopathy found a positive relationship between patients’ fear of panic-related physical symptoms and handicap (20,26). A recent cross-sectional study (27) measured dizziness-specific cognitions using the Illness Perceptions Questionnaire—Revised (IPQ-R) (28). This study found that negative perceived consequences of dizziness were the strongest correlate of dizziness handicap after adjusting for demographic variables, severity of symptoms, depression, and anxiety. This suggests beliefs about illness may be more important predictors of disability than anxiety, mood, and severity of symptoms, but longitudinal research is needed to confirm this relationship.

In a precursor to the current study, we found that levels of handicap and symptom severity measured with self-report questionnaires before attending a specialist dizziness clinic were not correlated with either health care professional assessed vestibular function or diagnoses (29). In contrast, psychological factors including distress (anxiety and depression), illness perceptions, and cognitive-behavioral responses to dizziness such as avoidance of activity and focusing on symptoms were significantly correlated with handicap and severity of symptoms. The addition of cognitive-behavioral symptom interpretations is important because interpretations of symptoms may be direct drivers of day-to-day behavior in people with vertigo and dizziness, which may ultimately lead to handicap. Psychological factors accounted for 53% and 30% of the variance in handicap and symptoms. There is therefore accumulating evidence for a range of common transdiagnostic psychological factors or mechanisms that might contribute to dizziness/vertigo-related disability.

The purpose of the current study was to extend the cross-sectional research by investigating longitudinally and prospectively whether this broader range of psychological factors and responses to symptoms before specialist input are associated with self-reported dizziness handicap 3 months after consultation and diagnosis. This article aims to answer the following questions:

1. Do dizziness handicap, distress, illness perceptions, and cognitive-behavioral responses to symptoms improve after specialist clinical assessment and diagnosis?
2. Are diagnostic category and vestibular test outcomes associated with self-reported handicap 3 months after consultation?
3. Do prediagnosis perceptions of dizziness, cognitive-behavioral responses to symptoms, and emotional factors predict handicap at 3 months after diagnosis?

## METHODS

### Participants

**FIGURE 1:**
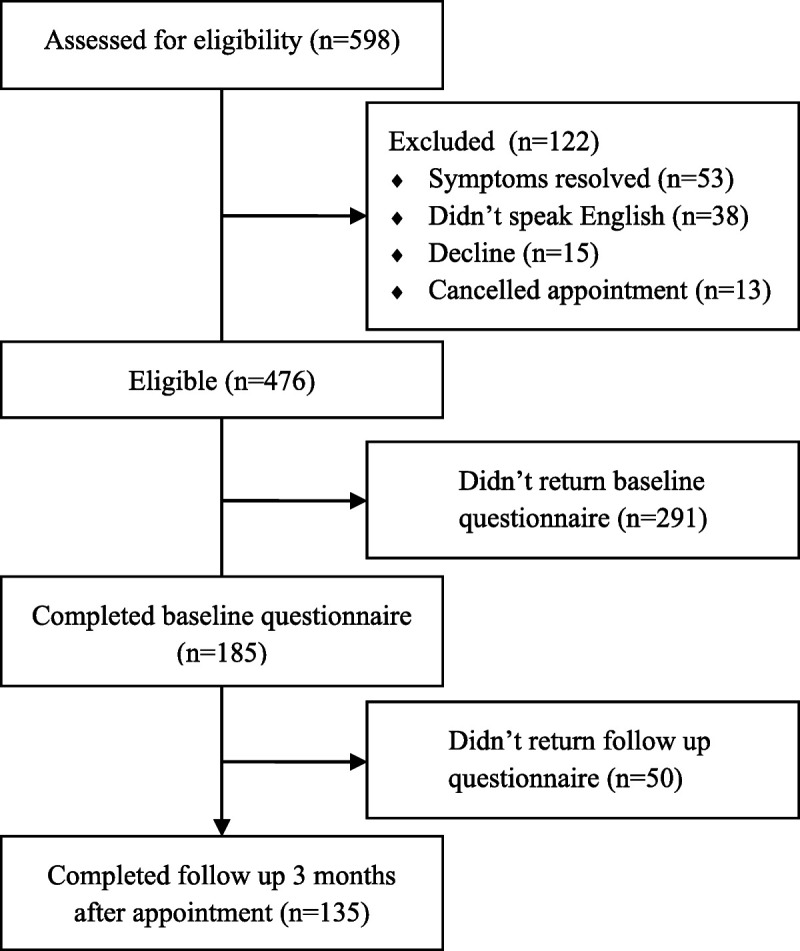
Participant flowchart.

Consecutive participants were recruited from the waiting list of the multidisciplinary balance clinic at Guy’s & St Thomas’ NHS Foundation Trust, London, between March and December 2018. Of the 476 eligible patients eligible to participate, 185 completed the baseline questionnaire and were contacted again after 3 months (Figure 1). The original cross-sectional findings are presented elsewhere (29). The study was approved by the NHS Health Research Authority (16/NI/0256).

### Data Collection

People on the waiting list received the questionnaire approximately 1 to 2 months before their initial appointment and completed it either electronically or via mail before they came for their appointment. Participants completed follow-up questionnaires 3 months after their initial diagnostic appointments. Reminders were sent out to nonresponders after 1 month. To facilitate follow-up, £10 expenses were sent to participants on completion of the three-month questionnaires.

### Measures

#### Primary Outcome

DHI (10) is a 25-question scale that measures the extent dizziness causes physical, functional, and emotional disability. Higher scores represent higher levels of handicap and activity restriction.

### Predictors

- Patient Health Questionnaire (PHQ-9) (30) is a nine-item scale that measures the frequency of depressive symptoms in the last 2 weeks from “0” (not at all) to “3” (nearly every day). Scores of 10 or more indicate probable depression.
- Generalized Anxiety Disorder (GAD-7) (31) is a seven-item scale that measures the frequency of anxiety symptoms in the last 2 weeks in the same way as the PHQ-9 and also has a cutoff of 10 or more for probable anxiety. For the purposes of analyses, it is also possible to combine the PHQ-9 and GAD-7 to form the Patient Health Questionnaire Anxiety and Depression Scale (32) as a composite measure of depression and anxiety.
- IPQ-R (28) measures illness-related cognitions (beliefs). In accordance with the author’s recommendations, the word “illness” was replaced with “dizziness condition” and the illness identity scale was modified to include symptoms relevant to people with vestibular disorders. The first domain measured the number of symptoms that the individual ascribed to their condition (*illness identity*). The other subscale measured how long they thought it would last (*timeline*), whether it would result in serious consequences (*consequences*), whether they believed they had power to influence their condition (*personal control*) or whether any treatment could improve it (*treatment control*), whether they understood the condition (*illness coherence*), whether the dizziness would come and go (*cyclical timeline*), and whether they had a strong emotional reaction when thinking about their dizziness (*emotional representation*). Participants are asked to respond to several statements for each domain on a 5-point Likert scale from “strongly agree” to “strongly disagree.”
- Cognitive-Behavioral Response to Symptoms Questionnaire (33) measures patients’ cognitive and behavioral responses to symptoms. The five subscales dealing with cognitive responses are symptom focusing, catastrophizing, damaging beliefs, fear avoidance, and embarrassment avoidance. The two behavioral subscales are all-or-nothing and avoidance/rest.
- Beliefs About Emotions Scale (34) measures the extent to which patients believe it is unacceptable to experience negative emotions or to express emotion to others.
- Psychological Vulnerability Scale (35) measures maladaptive cognitive responses related to perceptions of dependency, perfectionism, negative attributions, and the need for external sources of approval.

### Clinical Assessment and Treatment

All patients underwent a standardized clinical history and examination followed by a comprehensive vestibular battery to assess both peripheral and central vestibular function to reach a diagnosis. Findings for every patient were reviewed by the consultant audiovestibular physician (L.M.) who made the diagnosis based on consensus diagnostic criteria and commonly accepted definitions of the *International Classification of Vestibular Disorders* (36).

Vestibular function was assessed using the video head impulse test (vHIT), caloric irrigation, and videonystagmography, which are the main laboratory tests that measure different frequency functions of the vestibular organ, its reflexes, and central neural connections (37). Patients underwent further testing (such as imaging or vestibular-evoked myogenic potentials) or had additional examinations when clinically indicated to reach a final diagnosis. Further information on the vestibular testing can be found in the cross-sectional article (29).

Treatment of benign paroxysmal positional vertigo (BPPV) was carried out on the day; otherwise, patients were referred to see a physiotherapist for vestibular rehabilitation and/or the audiovestibular physician to discuss medical investigation or management. Patients also underwent psychological screening by validated questionnaires, and psychological assessment was recommended if they scored above the relevant threshold. The waiting lists to begin these treatments (other than BPPV) were typically longer than 3 months, although some patients may have been in the early stages of a vestibular rehabilitation program.

### Statistical Analysis

Data were analyzed using SPSS version 25. Two-sample *t* tests, χ^2^ test, and Fishers exact tests were used to examine the differences between responders and nonresponders. Because duration of dizziness was not normally distributed, this was log transformed for analyses. *t* Tests and analyses of variance (ANOVAs) explored the differences in handicap and psychological profile according to vestibular testing status and diagnoses, respectively. Paired-sample *t* tests showed the change in scores between baseline and follow-up. Bivariate Pearson correlations explored the relationship between the psychological variables and handicap and partial correlations adjusted for baseline handicap. Because of multiple tests, we used the more stringent *p* < .001 to interpret relationships as significant. To assess if type of diagnosis affected the results, χ^2^ tests were performed. Hierarchical multiple linear regression was performed to predict DHI at follow-up. A dummy variable for vestibular testing was created to account for whether patients had any evidence of vestibular abnormality on one or more laboratory tests, consistent with diagnostic approaches in the internationally accepted diagnostic criteria of the Barany Society (36).

## RESULTS

### Participants

One hundred eighty-five consecutive patients completed the baseline questions, and 135 (73%) returned completed questionnaires at 3-month follow-up (Figure 1). There were no significant differences between responders (*n* = 135) and dropouts (*n* = 50) for the demographic variables or primary diagnosis (Table 1). For responders, the mean (standard deviation [SD]) duration of illness at baseline was 50.57 (69.161) months, and the median was 24 months (Table 1). Of all the demographic variables and diagnoses, only duration of dizziness at baseline was correlated with handicap at follow-up (*r* = 0.28, *p* = .001).

**TABLE 1:** Demographic and Diagnoses for Responders at Baseline and Follow-Up

Baseline Variable	Respondents at Baseline (*n* = 185)	Respondents at 3 mo (*n* = 135)	Statistical Comparison
Age, y			
M (SD)	53.57 (17.386)	54.23 (17.531)	*t* = −0.850, *p* = .40, 95% CI = −8.133 to 3.233
Range	18–90	18–90	
Sex: female, *n* (%)	137 (74.1)	98 (72.6)	χ^2^ = 0.555, *p* = .46
Duration, mo			
M (SD)	48.33 (64.359)	50.57 (69.161)	*U* = 3569.5, *p* = .55
Median	24	24	
Ethnicity, *n* (%)			χ^2^ = 1.775, *p* = .18
White	152 (82)	114 (84.4)	
Black, minority ethnic	33 (18)	21 (15.6)	
Marital status, *n* (%)			χ^2^ = 2.656, *p* = .75
Married/civil partnership	69 (37.3)	53 (39.3)	
Living with partner	28 (15.1)	19 (14.1)	
Single	48 (25.9)	35 (25.9)	
Divorced	19 (10.3)	15 (11.1)	
Separated	7 (3.8)	4 (3)	
Widowed	14 (7.6)	9 (6.7)	
Employment*, n* (%)			χ^2^ = 6.163, *p* = .41
Employed (full time)	55 (29.7)	37 (27.4)	
Employed (part time)	21 (11.4)	15 (11.1)	
Unemployed	26 (14.1)	18 (13.3)	
Retired	58 (31.4)	48 (35.6)	
Student	6 (3.2)	5 (3.7)	
Home maker	5 (2.7)	4 (3.0)	
Other	14 (7.6)	8 (5.9)	
Education, *n* (%)			χ^2^ = 5.059, *p* = .65
Postgraduate	28 (15.1)	6 (2.2)	
University	48 (25.9)	33 (24.4)	
Trade/apprenticeship	8 (4.3)	7 (5.2)	
Certificate/diploma	24 (13)	17 (12.6)	
A-levels	14 (7.6)	11 (8.1)	
GCSE	35 (18.9)	29 (21.5)	
No formal education	20 (10.8)	12 (8.9)	
Other	8 (4.3)	6 (4.4)	
Diagnosis, *n* (%)			χ^2^ = 4.467, *p* = .88
UPV	45 (25.4)	34 (25.2)	
BPPV	37 (20.9)	27 (20)	
VM	38 (21.5)	28 (20.7)	
Functional (e.g., PPPD)	11 (6.2)	7 (5.2)	
MD	9 (5.1)	6 (4.4)	
Central	5 (2.8)	5 (3.7)	
BPV	5 (2.8)	4 (3)	
Vestibular schwannoma	3 (1.7)	3 (2.2)	
SSCD	2 (1.1)	1 (0.7)	
Other	22 (12.4)	16 (11.9)	

M (SD) = mean (standard deviation); GCSE = General Certificate of Secondary Education; UPV = unilateral peripheral vestibulopathy; BPPV = benign paroxysmal positional vertigo; VM = vestibular migraine; PPPD = persistent postural perceptual dizziness; MD = Meniere disease; Central = central nervous system disorders; BPV = bilateral peripheral vestibulopathy; SSCD = superior semicircular canal dehiscence.

### Do Dizziness Handicap, Distress, Illness Perceptions, and Cognitive-Behavioral Responses to Symptoms Improve After Clinical Assessment and Diagnosis?

#### Change in Handicap Scores

Baseline handicap measured by DHI was strongly correlated with handicap at 3-month follow-up (*r* = 0.83, *p* < .01). There was a mean (SD) improvement of 7.45 (14.57), which was statistically significant (*t*(134) = 5.944, *p* < .001). The maximum improvement was 52, and the maximum deterioration was 34. According to the clinically meaningful change score of 18 points as described by Jacobson and Newman (10), 3% (*n* = 4) of participants worsened, 75% (*n* = 101) stayed the same, and 22% (*n* = 30) improved. According to the recommended cutoffs, 39% (*n* = 52) had mild handicap, 36% (*n* = 49) had moderate, and 18% (*n* = 34) had severe handicap at follow-up.

### Change in Psychological Factors

#### Change in Anxiety and Depression Scores

There was a mean (SD) improvement from baseline to 3 months of 1.05 (4.97) on the depression scale (PHQ-9), which was statistically significant (*t*(134) = 2.459, *p* = .015). There was also a significant mean (SD) improvement of 1.04 (4.91) on the anxiety scale (GAD-7; *t*(134) = 2.454, *p* = .015). At 3 months, the proportion of participants who scored above the clinical threshold for suspected depression and anxiety remained the same (Table 2). At baseline, 41% (*n* = 55) had at least one measure of distress that met the cutoff compared with 37% (*n* = 50) at follow-up.

**TABLE 2:** Number of Participants Meeting Cutoff Scores for Distress Measures

	Baseline	Follow-up
Depression (PHQ-9 ≥ 10)	51 (38%)	43 (32%)
Anxiety (GAD-7 ≥ 10)	34 (25%)	33 (24%)
No. distress measures meeting cutoff		
1	25 (19%)	24 (18%)
2	30 (22%)	26 (19%)

PHQ-9 = Patient Health Questionnaire, GAD-7 = Generalized Anxiety Disorder Scale.

#### Change in Illness Perceptions

When compared with baseline, at 3 months after diagnosis, participants had significantly greater understanding (coherence) of their condition, considered dizziness to have less serious consequences to their lives and had reduced negative emotions in relation to the condition (Table 3). For belief in the chronic or cyclical nature of their condition, and personal and treatment control, there were no significant differences in scores. Participants attributed fewer symptoms to their condition (illness identity) at follow-up, although the significance was borderline (*p* = .053).

**TABLE 3:** Comparison of IPQ-R and CBRSQ Scores at Baseline and Follow-Up

	Baseline, M (SD)	Follow-Up, M (SD)	Paired *t* Test (95% CI)
IPQ-R			
Illness identity	9.73 (5.24)	8.84 (5.42)	*t* = 1.951, *p* = .053 (−0.012 to 1.790)
Timeline (chronic)	18.94 (4.79)	18.45 (5.12)	*t* = 1.351, *p* = .18 (−0.228 to 1.210)
Timeline (cyclical)	13.98 (3.69)	13.52 (3.73)	*t* = 1.351, *p* = .18 (−0.213 to 1.132)
Consequences	18.83 (5.67)	17.68 (5.75)	*t* = 2.714, *p* = .008 (0.311 to 1.982)
Emotional representations	20.35 (5.79)	18.64 (6.10)	*t* = 4.147, *p* < .001 (0.895 to 2.527)
Personal control	17.29 (4.40)	17.70 (4.41)	*t* = −1.072, *p* = .29 (−1.159 to 0.344)
Treatment control	16.11 (3.47)	16.28 (4.41)	*t* = 4.464, *p* = .64 (−0.907 to 0.562)
Illness coherence	11.60 (4.62)	14.24 (5.07)	*t* = −5.83, *p* < .001 (−3.534 to −1.744)
CBRSQ			
Symptom focusing	19.17 (5.765)	17.53 (6.374)	*t* = 3.512, *p* = .001 (0.718 to 2.571)
Catastrophizing	11.41 (3.946)	10.32 (4.001)	*t* = 4.174, *p* < .001 (0.577 to 1.616)
Damaging beliefs	15.21 (4.074)	13.84 (3.961)	*t* = 4.959, *p* < .001 (0.824 to 1.917)
Fear avoidance	18.53 (4.731)	17.04 (4.893)	*t* = 4.058, *p* < .001 (0.763 to 2.215)
Embarrassment avoidance	15.82 (6.268)	14.56 (6.752)	*t* = 3.122, *p* = .002 (0.461 to 2.057)
All-or-nothing behavior	11.56 (5.288)	11.03 (5.081)	*t* = 1.488, *p* = .14 (−0.173 to 1.225)
Avoidance/resting behavior	18.63 (7.068)	17.50 (7.244)	*t* = 2.507, *p* = .013 (0.239 to 2.028)

IPQ-R = Illness Perceptions Questionnaire—Revised; CBRSQ = Cognitive-Behavioral response to Symptoms Questionnaire; M (SD) = mean (standard deviation); CI = confidence interval.

At follow-up, 56% of participants had not changed their symptom attribution. Twenty-three percent of participants adopted a more psychological attribution, and 21% adopted a more physical attribution for their symptoms. Despite this individual variation in symptom attribution, a McNemar test determined that the difference in the proportion of participants with physical, psychological, and combined attributions at baseline and follow-up was not significantly different (χ^2^(3) = 5.032, *p* = .17).

#### Change in Cognitions and Behavioral Responses to Symptoms

There was a significant improvement in all the symptom cognitions (Table 3). Avoidance behavior also significantly improved, although all-or-nothing behavior did not significantly change.

### Are Type of Diagnosis and Audiovestibular Test Outcomes Associated With Self-Reported Handicap 3 months After Consultation?

A one-way Welch ANOVA was conducted to determine if the level of handicap was different for the top 5 diagnostic groups, as the assumption of homogeneity of variances was violated (Levene test, *p* = .047). DHI scores increased from people with the Meniere disease (M [SD] = 29 [20]), to functional dizziness (M [SD] = 31 [23]), to vestibular migraine (M [SD] = 40 [29]), to BPPV (M [SD] = 41 [32]), to chronic unilateral peripheral vestibulopathy (M [SD] = 42 [21]), in that order. However, the overall test of differences between the groups was not statistically significant (Welch’s *F*(4, 21.763) = 0.690, *p* = .61).

An independent-samples *t* test was run to determine if there were differences in handicap between patients with and without vestibular deficits. Patients with normal vestibular function scored 2.7 points (95% confidence interval [CI], −6.49 to 11.89) higher than did patients with abnormal vestibular function, which was not significant (*t*(128) = 5.81, *p* = .56). There were no significant differences in dizziness handicap at follow-up when each of the most frequently completed vestibular tests was analyzed individually, which included videonystagmography (M = 2.92, 95% CI, −9.837 to 15.676, *t*(125) = 0.453, *p* = .65), vHIT (M = −1.03, 95% CI = −15.73 to 13.66, *t*(109) = −0.139, *p* = .89), and caloric paresis (M = −4.254, 95% CI = −14.209 to 5.701, *t*(91) = −0.849, *p* = .40).

### Do Prediagnosis Perceptions of the Dizziness, Cognitive-Behavioral Responses to Symptoms and Emotional Factors Predict Handicap at 3 Months After Diagnosis?

#### Dizziness Handicap: Bivariate Correlations

Table 4 shows correlations for the psychological variables measured at baseline with the dizziness handicap score at 3 months. Most baseline variables showed moderate to large associations with handicap at 3 months. Baseline anxiety and depression, all of the subscales of the Cognitive-Behavioral Response to Symptoms Questionnaire, and the identity, chronic timeline, serious consequences, and emotional representation subscales of the IPQ-R were all significant correlates (*p* < .001) of handicap at 3 months. The personal control, treatment control, illness coherence, and cyclical timeline subscales of the IPQ-R and the beliefs about emotions scale were not significantly related to handicap. Partial correlations after adjusting for baseline handicap reduced the correlations to nonsignificant except for all-or-nothing behavior, which continued to be independent predictors of higher levels of handicap at 3 months.

**TABLE 4:** Correlations Between Baseline Psychological Variables and Dizziness Handicap at Follow-Up

Psychological Variables at Baseline	Dizziness Handicap at 3 mo	
	Correlation (*r*)	Partial Correlations (*r*) Controlling for Baseline Handicap
Psychological distress		
Depression (PHQ-9)	0.675*	0.244
Anxiety (GAD-7)	0.594*	0.192
CBRSQ		
Symptom focusing	0.392*	0.184
Catastrophizing	0.512*	0.145
Damaging beliefs	0.368*	0.066
Fear avoidance	0.411*	0.008
Embarrassment avoidance	0.594*	0.148
All-or-nothing behavior	0.498*	0.289*
Avoidance/resting behavior	0.605*	0.196
IPQ-R		
Identity	0.387*	0.088
Chronic timeline	0.366*	0.192
Consequences	0.539*	0.062
Personal control	−0.003	0.031
Treatment control	−0.205	−0.068
Illness coherence	0.069	0.132
Cyclical timeline	0.141	0.183
Emotional representation	0.478*	0.164
Beliefs About Emotions	0.027	0.074
Psychological Vulnerability	0.347*	0.189

PHQ-9 = Patient Health Questionnaire, GAD-7 = Generalized Anxiety Disorder Scale; CBRSQ = Cognitive Behavioural response to Symptoms Questionnaire; IPQ-R = Illness Perceptions Questionnaire—Revised.

Partial correlation for association between baseline psychological variables and dizziness handicap at 3-month follow-up, controlling for dizziness handicap at baseline.

* *p* < .001.

### Regression

Hierarchical multiple linear regression was performed to predict DHI at follow-up (3 months), entering age, sex, and baseline DHI as control variables followed by baseline psychological variables, which were correlated with DHI at follow-up, with a correlation of ≥0.2 (Table 5). Because of collinearity, the emotional representation variable of the IPQ-R was excluded from the analyses as it overlaps with depressive symptoms and PHQ-9 and GAD-7 scores were grouped (Patient Health Questionnaire Anxiety and Depression Scale) as a composite measure of distress (33). This model was significant (adjusted *R*^2^ = 0.735, ANOVA *F* = 22.86, *p* < .001) in which baseline dizziness handicap explained the most variance in handicap at follow-up, although adding the psychological variables still contributed an additional significant 3% to the model.

**TABLE 5:** Regression Model of DHI at Follow-Up (3 mo)

Predictors (Baseline)	Dizziness Handicap (Follow-Up)				
	Δ*R*^2^	Std. Error of the Estimate	*R*^2^ Change	*F* Change	Sig. *F* Change
Step 1					
Demographic variables*^a^*	0.082	25.112	0.103	4.987	.003
Step 2					
Baseline DHI	0.707	14.186	0.613	280.520	.000
Step 3					
Psychological variables*^b^*	0.735	13.492	0.053	2.056	.022

DHI = Dizziness Handicap Inventory.

*^a^* Control variables included age, sex, and duration of symptoms.

*^b^* Psychological variables included The Patient Health Questionnaire Anxiety and Depression Scale, the Psychological Vulnerability, and subscales from the Illness Perceptions Questionnaire—Revised and Cognitive-Behavioral Response to Symptoms Questionnaire.

## DISCUSSION

The study found a significant improvement in dizziness handicap, anxiety, and depression 3 months after an initial consultation in a specialist vestibular clinic. Participants perceived fewer negative consequences, had significantly greater understanding of their illness, and were less emotionally affected by their condition. Participants also reported reductions in unhelpful cognitive-behavioral responses to symptoms (e.g., less symptom focusing, catastrophizing about symptoms, and avoiding activities because of embarrassment and/or fear). There was no change in all-or-nothing (“boom-bust”) behavior. Dizziness handicap at follow-up was associated with symptom duration, but not with any other demographic factor, diagnosis, or vestibular function test. The baseline self-report psychological measures were associated with dizziness handicap at follow-up, although the correlations were no longer significant after adjusting for baseline dizziness handicap except for all-or-nothing behavior in response to symptoms. The fully adjusted model explained 74% of the variance in dizziness handicap at follow-up with the psychological factors explaining a significant 3%, and baseline dizziness handicap explaining the majority of the variance.

The data suggest that, although there was a significant improvement in handicap after diagnosis, the change was small and self-reported handicap remained relatively stable over the 3-month period of the study. Although the psychological measures improved, the overall levels indicated that participants still tended to view their condition negatively and the rates of illness distress remained elevated. These participants had received a diagnosis and some treatment, and although these seem to reduce handicap, more is clearly needed to reduce handicap further as many patients were still significantly impaired.

There was no difference in self-reported handicap between the most common diagnoses and between patients with and without evidence of structural vestibular dysfunction. Normally, vestibular reflex function is highly correlated with vestibular perception. For example, when the vestibular system is stimulated (e.g., by irrigating the ear canal with warm water in the case of the “caloric test”), there will be a vestibular ocular reflex response manifest as a spontaneous eye movement (called nystagmus) and reproduction of vertigo. However, standard laboratory tests of vestibular reflex function seem to tell us little of how the patients with chronic disorders are feeling or their daily functioning.

These findings are in accordance with previous findings that neither caloric nor vHIT results predict symptom outcome in vestibular neuritis (13). Allum et al. (38) also demonstrated that recovery occurs both in patients who recover peripheral (caloric) vestibular function and in those who do not. This occurs because of brain plasticity, which is influenced by exposure as observed in individuals who adapt to repeated vestibular stimulating from training (dancers) (39). Neuroimaging studies have also identified a wider vestibular network in the brain (7) that goes beyond the traditional, lower-level reflex motor circuits measured using standard laboratory testing. These studies have started to find correlates between handicap and vestibular functional architecture (40) that may help us understand further the relationships between physiology and ongoing symptoms and handicap.

Although more work is needed to understand the biology underpinning ongoing symptoms, the results do point to a number of possible mechanisms that may contribute to the perpetuation of dizziness handicap. The most important predictor in this study appeared to be “all-or-nothing” (or “boom-bust”) behavioral responses to symptoms, which was the only item to retain its association with dizziness handicap over time when baseline dizziness was adjusted. This may be because people who engage in this behavior tend to be quite symptom contingent, so if they are feeling good, they may overdo activity and then crash. This may lead to future negatively conditioned emotional responses to physical activity and dizziness.

Although other psychological variables were correlated with handicap over time, their effect on handicap disappeared when adjusting for baseline handicap. These factors were also relatively stable overtime, so it may be that they contribute to a vicious cycle of handicap whereby understandable cognitive behavioral and emotional responses to the initial symptoms and handicap actually contribute to the severity of the symptoms overtime.

For example, anxiety and depression could influence self-perceived handicap in a number of ways. Anxiety arousal can increase the somatic symptoms induced by balance disorders (15) and exert direct effects on vestibular information processing required for the perception and control of orientation (22). Anxiety and negative affect are also closely related to reporting of physical symptoms and negative attributional processes that can contribute to an escalating cycle of conditioned fear, arousal, and restriction of activity (41).

The relationship between distress and handicap, however, is imperfect. To adapt successfully to long-standing dizziness and vertigo, people also need to develop relatively accurate and balanced beliefs about symptoms, illness, and treatment. People develop their own “common sense” model of their condition, and sometimes that can be more negative than it needs to be or less accurate in some way (42,43). In this study, participants who attributed a wider range of symptoms to their condition (higher illness identity), believed that their condition would last a long time, have more serious consequences, and be less likely to respond to treatment had higher levels of dizziness handicap at follow-up.

In their cross-sectional study, Wolf et al. (27) also found correlations between handicap and illness perceptions, particularly negative perceived consequences. In this study, some but not all of the illness perceptions improved at follow-up, suggesting that somatic experience early in the temporal sequence of the condition is important in the development and maintenance of negative illness perceptions. It is important to note than in some instances, patients’ symptom interpretations may indeed be accurate, but the overriding tendency to view symptoms of dizziness negatively seems to be unhelpful. Therefore, it is important to explore how patients think about or understand their condition and have some idea of whether that is an accurate or balanced view or not.

It is not only the overall representation of the illness that is important but also the day-to-day interpretation of symptoms, which seemed to be more consistently associated with self-reported handicap than beliefs about the illness as a whole. This may be because patients tend to focus on the symptoms rather than on more sophisticated or complex representations of their condition (44). Dizziness handicap was higher in patients who focused more on their symptoms, catastrophized about the consequences of experiencing symptoms, believed that their dizziness symptoms were a sign of physical damage, were fearful of activity, and felt embarrassed about their symptoms.

These findings add to previous research that show that patients with dizziness frequently endorse such negative beliefs (25), and that concerns of social embarrassment and being unable to fulfill normal roles contribute to dizziness handicap (17). Pothier et al. (45) also found that catastrophizing about dizziness explained a significant proportion of the variance in handicap after adjusting for mood. Thus, focusing on physical symptoms may effectively increase those sensations, and if patients perceive dizziness as a sign of imminent threat, their attempts to cope with this possibility may effectively prolong their handicap because restriction of physical movement may hinder natural recovery (compensation) from the initial vestibular dysfunction (46) and reinforce negative perceptions.

In this study, patients with greater psychological vulnerability at baseline and follow-up also reported more dizziness handicap. Psychological vulnerability refers to people who base their self-esteem and respect on input from or in relation to others (35). It could place patients at risk of greater distress and/or maladaptive coping, such as pressure to push on and then crash (all-or-nothing behavior), because of perceived failure to live up to certain high standards. This could be relevant to people with vestibular diseases because vertigo and dizziness symptoms can markedly interfere with one’s ability to achieve goals.

This study uniquely recorded data before diagnosis in a representative sample of patients attending a specialist clinic for dizziness. This was a pragmatic longitudinal design, and interpretation of causality is still limited owing to the imperfect control for confounders that exists outside a randomized trial and without multiple assessment points. Although we statistically adjusted for baseline dizziness handicap, this will not fully control for past exposure. Therefore, some reverse causality could be present in our estimates of the association between baseline variables and 3-month handicap. It was not possible to control for the treatment delivered to patients, although 3-month follow-up was chosen because most patients would have either not yet received treatment or been in the very early stages. It was also not possible to ascertain precisely when they completed the baseline measures before their diagnostic appointments.

Likewise, although the sample was representative, the response rate at follow-up may have affected our ability to detect more meaningful results, meaning some effects may have been underestimated. Nevertheless, our data point to the major relevance of longitudinal change in patients’ perceptions, cognitions, and behaviors, as well as their negative affect, in understanding their levels of dizziness handicap regardless of neuro-otological diagnosis or vestibular function status.

## CONCLUSIONS

Psychological factors including distress, dizziness-specific cognitions, behavioral responses, and negative illness perceptions before attending a specialist neuro-otology clinic predict ongoing dizziness handicap. The diagnostic process was associated with improvements in dizziness and psychological factors, although the level of distress remained high. Patients still tended to view their condition in a negative way and exhibit unhelpful cognitive and behavioral responses to symptoms. Vestibular function tests or diagnosis, on the other hand, were not associated with ongoing dizziness handicap. Future studies should investigate whether targeting these factors alongside vestibular rehabilitation improves handicap in patients with chronic dizziness.
